# LOC100912399 regulates osteogenic differentiation of bone marrow mesenchymal stem cells through modulating p38MAPK signaling-mediated oxidative stress and apoptosis

**DOI:** 10.1038/s41598-026-45292-9

**Published:** 2026-04-01

**Authors:** Cheng-Song Lan, Pao Wang, Teng Kang, Hao Qin, Zhi-Wei Liu, Sheng-Jie Gu, Jing-Peng Zhang, Gang Liu

**Affiliations:** 1 Emergency Department, The Affiliated Hospital of Guizhou Medical University, Guiyang, 550004 Guizhou China; 2 Department of Orthopedics, People’s Hospital of Dejiang, Tongren, 565200 Guizhou China; 3 Department of Orthopedics, The Fourth People’s Hospital of Guiyang, Guiyang, 550007 Guizhou China; 4 Department of Orthopedics, Xingyi People’s Hospital, Xingyi, 562499 Guizhou China

**Keywords:** Long non-coding RNA, Bone marrow mesenchymal stem cells, Osteogenic differentiation, Oxidative stress, p38MAPK, Cell biology, Molecular biology, Stem cells

## Abstract

This study aims to investigate the role and underlying mechanism of the long non-coding RNA (lncRNA) LOC100912399 in regulating osteogenic differentiation of bone marrow mesenchymal stem cells (BMSCs), with a specific focus on its involvement in oxidative stress-induced apoptosis via the p38 mitogen-activated protein kinase (p38MAPK) signaling pathway. BMSCs were isolated from the tibiae and fibulae of Sprague-Dawley (SD) rat pups and cultured to the third passage. Cell purity was confirmed by flow cytometric analysis of surface markers (CD45, CD29, CD106, CD90, CD11b), and multilineage differentiation potential was verified using Alizarin Red S (osteogenic), Oil Red O (adipogenic), and Alcian Blue (chondrogenic) staining. At 90% confluence, BMSCs were transduced with lentiviral vectors for LOC100912399 overexpression (Lv-LOC100912399) or knockdown (Sh-LOC100912399), alone or in combination with p38MAPK pathway inhibition or overexpression (Lv-p38MAPK), followed by exposure to oxidative stress for 48 h. Apoptosis was evaluated by Annexin V/PI staining, cell viability by CCK-8 assay, and gene expression by quantitative real-time PCR (qRT-PCR). Protein levels of oxidative stress-related enzymes (MnSOD, CAT, GPx), apoptosis-related factors (Bcl-2, Bax, p-p38MAPK), and osteogenic markers (RUNX2, OPN, ALP) were determined by Western blot. High-purity BMSCs (> 99%) were successfully isolated and characterized. Overexpression of LOC100912399 significantly promoted oxidative stress-induced apoptosis, reduced cell viability, downregulated antioxidant enzyme expression, increased the Bax/Bcl-2 ratio, and inhibited osteogenic differentiation (*P* < 0.05). Conversely, knockdown of LOC100912399 enhanced antioxidant capacity, suppressed apoptosis, and upregulated osteogenic marker expression (*P* < 0.05). These effects were further modulated by either p38MAPK overexpression or inhibition, indicating that LOC100912399 functions, at least in part, through the p38MAPK signaling pathway. LOC100912399 regulates oxidative stress and apoptosis in BMSCs via the p38MAPK signaling pathway, thereby influencing osteogenic differentiation. Targeting LOC100912399 may represent a potential therapeutic strategy for enhancing bone formation under oxidative stress-related conditions such as osteoporosis.

**Supplementary Information:**

The online version contains supplementary material available at 10.1038/s41598-026-45292-9.

## Introduction

## Oxidative stress: a pivotal pathogenic factor in osteoporosis

Under physiological conditions, intracellular oxidative and antioxidant systems maintain a delicate dynamic balance. Oxidative stress (OS) refers to a pathophysiological state characterized by the disruption of redox homeostasis in response to adverse stimuli^1^. This imbalance arises through two primary mechanisms: excessive production of reactive oxygen/nitrogen species (ROS/RNS), including superoxide anions (O_2_•⁻), hydroxyl radicals (•OH), and nitric oxide (NO), coupled with impaired function of antioxidant defense systems—encompassing enzymes such as manganese superoxide dismutase (MnSOD), catalase (CAT), and glutathione peroxidase (GPx), as well as non-enzymatic antioxidants (e.g., vitamins E, C, and A)^2^. The consequent redox dysregulation induces macromolecular damage, genomic instability, lipid peroxidation, mitochondrial dysfunction, and activation of apoptotic pathways, ultimately leading to cellular death and tissue injury^3^. Notably, OS has been implicated as a critical pathogenic factor in numerous diseases, including malignancies, cerebrovascular accidents, osteonecrosis, myocardial infarction, and neurodegenerative disorders^4^.

Accumulating evidence highlights the pivotal role of OS in the pathogenesis of osteoporosis (OP), a systemic metabolic bone disorder characterized by uncoupled bone remodeling—diminished osteoblastic bone formation and enhanced osteoclastic bone resorption—resulting in reduced bone mass, deteriorated bone microarchitecture, and increased fracture risk^5,6^. As a growing global health concern, OP-related complications (e.g., chronic pain, fragility fractures, spinal deformities) significantly compromise quality of life^7^. Clinical studies have shown elevated OS biomarkers in OP patients compared to age-matched healthy controls, underscoring the contribution of oxidative damage to disease progression^8^. While estrogen deficiency remains a well-established driver of OP, recent investigations have revealed that sex hormones exert antioxidant effects by neutralizing pro-oxidants and promoting osteoblast survival, establishing an intricate link between hormonal regulation and oxidative balance in bone metabolism^9,10^.

## LncRNAs as key regulators in skeletal pathologies and osteoporosis

Long non-coding RNAs (lncRNAs) are defined as transcripts exceeding 200 nucleotides in length with no protein-coding capacity. They have emerged as key regulators of cellular differentiation and gene expression networks^11^. Dysregulation of lncRNAs—through mutation, aberrant expression, or deletion—contributes to various skeletal pathologies, including bone tumors, osteoarthritis, and OP^12^. In the context of OP, lncRNAs modulate critical cellular processes in bone-related cells, such as proliferation, differentiation, apoptosis, and oxidative stress responses^13^.

## The p38MAPK signaling pathway in bone homeostasis and stress responses

Among the signaling pathways governing bone homeostasis, the mitogen-activated protein kinase (MAPK) cascade—particularly the p38MAPK subfamily—plays a central role in mediating stress responses, inflammation, and apoptosis^14,15^. The p38α (MAPK14) isoform acts as a critical regulator of apoptosis through rapid phosphorylation-dependent activation^16,17^. The precise spatiotemporal regulation of this pathway makes it a key determinant of cellular fate under stress conditions^18^. Intriguingly, multiple lncRNAs have been shown to interact with components of the MAPK signaling pathway, suggesting functional crosstalk in the modulation of apoptosis^19^.

## Rationale and hypothesis of the present study

 Our preliminary data demonstrated significant upregulation of LOC100912399 (annotated near the MAP3K1 locus) in BMSCs exposed to oxidative stress. This led us to hypothesize that LOC100912399 regulates OS-induced apoptosis via the p38MAPK signaling pathway, thereby influencing osteogenic differentiation. The present study aims to elucidate this novel mechanism, providing insights into the pathogenesis of OP and identifying potential therapeutic targets.

## Materials and methods

### Methodology validation statement

All experiments in this study were conducted in accordance with the guidelines of the Ethics Committee of Guizhou Medical University. Ethical approval was granted prior to the initiation of the study from the Ethics Committee of Guizhou Medical University (Approval No. 2304021; Date: March 02, 2023).

Bone marrow mesenchymal stem cells (BMSCs) were isolated, cultured, and purified from the tibiae and fibulae of Sprague-Dawley (SD) rats. Cell identity was confirmed by the detection of surface antigen markers (CD45, CD29, etc.) and verification of multilineage differentiation potential (osteogenic, adipogenic, chondrogenic). Third-passage BMSCs (at 90% confluence) were subjected to the following treatments: LOC100912399 overexpression (Lv-LOC100912399), p38MAPK inhibition, or LOC100912399 knockdown (Sh-LOC100912399) combined with p38MAPK overexpression. Subsequent to these treatments, cells were exposed to oxidative stress for 48 h. The expression levels of LOC100912399 and p38MAPK were assessed by quantitative polymerase chain reaction (qPCR). Apoptosis was detected via Annexin V/PI staining, cell proliferation was evaluated using the CCK-8 assay, and the protein expression levels of oxidative stress-related proteins (MnSOD, CAT, GPx), apoptosis-related proteins (Bcl-2, Bax, p38MAPK), and osteogenic-related proteins (RUNX2, OPN, ALP) were measured by Western blot. Statistical analysis was performed using the SPSS 23.0 software package with one-way analysis of variance (ANOVA), and a *P*-value < 0.05 was considered statistically significant.

### Extraction, culture, and resuscitation of rat BMSCs

Healthy SD rats (2–3 weeks old, 20–30 g, both sexes) were used in this study. Rats were euthanized by cervical dislocation, soaked in 75% ethanol for 10 min, and their lower limbs were dissected under sterile conditions. The tibiae and femora were completely isolated, soft tissues were thoroughly removed, and the epiphyses were excised. The bone marrow cavities were flushed with complete culture medium until transparent, and the flushing fluid was collected and centrifuged at 1000 rpm for 5 min. The supernatant was discarded, and the cell pellet was resuspended in low-glucose complete medium. The cell suspension was transferred to T25 cm^2^ culture flasks and incubated at 37 °C in a humidified atmosphere containing 5% CO_2_. A half-volume medium change was performed at 24 h after seeding, followed by full medium changes every 2–3 days thereafter. When cell confluence reached approximately 90%, routine subculture was carried out. For cell resuscitation, frozen target cells were retrieved from the − 80 °C freezer and thawed in a 37 °C water bath. The thawed cells were centrifuged, the supernatant was discarded, and the cell pellet was resuspended in complete medium before being transferred to T25 cm^2^ culture flasks for continued culture.

### Identification of BMSC surface markers

BMSCs were digested with 0.25% trypsin-0.02% EDTA, and the cell density was adjusted to 2 × 10⁷ cells/mL. A 50 µL aliquot of the cell suspension was transferred to each Eppendorf (EP) tube. To each tube, 5 µL of primary antibodies against CD106, CD45, CD29, CD11b, or CD90 was added, followed by 45 µL of buffer. The tubes were incubated at room temperature for 20 min in the dark. After incubation, the cells were washed twice with incubation buffer, centrifuged to collect the cell pellet, and resuspended in 500 µL of incubation buffer for flow cytometric analysis.

#### CCK-8 assay

Cell suspensions were seeded into 96-well plates at a density of at least 5000 cells per 100 µL per well and incubated overnight. Experimental groups were cultured for 48 h (with 3 replicate wells per group). After incubation, 10 µL of CCK-8 solution was added to each well, and the plate was returned to the incubator for an additional 2 h. The absorbance (A) value at 450 nm was measured using a microplate reader.

#### Flow cytometric detection of oxidative stress-induced apoptosis in BMSCs

Cells were digested with 0.25% trypsin-0.02% EDTA, collected by centrifugation, and washed twice with phosphate-buffered saline (PBS). The cells were centrifuged at 2000 rpm for 5 min, and the pellet was resuspended in 1× Binding Buffer to a final concentration of 1 × 10^6^ cells/mL. A 100 µL aliquot of the cell suspension was transferred to 5 mL culture tubes, and 5 µL of FITC Annexin V and 5 µL of propidium iodide (PI) were added to each tube. The cells were gently vortexed and incubated at room temperature for 15 min in the dark. After incubation, 500 µL of 1× Binding Buffer was added to each tube, and the samples were analyzed by flow cytometry immediately.

### Western blot analysis

Target proteins were extracted from cells, and protein quantification was performed using the bicinchoninic acid (BCA) method. After determining the protein concentration of each group, the protein concentration of all samples was normalized using deionized water and protein loading buffer. Protein samples were then separated by sodium dodecyl sulfate-polyacrylamide gel electrophoresis (SDS-PAGE) and transferred onto polyvinylidene difluoride (PVDF) membranes. The membranes were blocked with blocking solution for 1 h at room temperature, followed by overnight incubation at 4 °C with primary antibodies against Bcl-2, Bax, p38MAPK, MnSOD, CAT, GPx, RUNX2, OPN, and ALP. On the following day, the membranes were washed with Tris-buffered saline with Tween 20 (TBST) and incubated with the corresponding secondary antibodies at room temperature for 1 h. The exposure solution was prepared at a 1:1 ratio, and the PVDF membranes were placed on filter paper to dry. The membranes were then placed in an exposure apparatus, the exposure solution was added, and protein bands were exposed and imaged. The relative expression level of each target protein was calculated based on the grayscale values of the target bands and the internal reference (β-actin) bands.

### Quantitative real-time PCR (qPCR)

Total RNA was extracted from cells and reverse-transcribed into complementary DNA (cDNA) using a reverse transcription kit in a 20 µL reaction volume. The specific amplification conditions were as follows: pre-denaturation at 95 °C for 2 min, followed by 40 cycles of denaturation at 95 °C for 10 s and annealing at 60 °C for 30 s. The relative expression levels of LOC100912399 and p38MAPK were calculated using the 2^(-ΔΔCT) method, with glyceraldehyde-3-phosphate dehydrogenase (GAPDH) serving as the internal reference gene. The primer sequences used were as follows:

- GAPDH: upstream 5’-GGAGCGAGATCCCTCCAAAAT-3’, downstream 5’-GGCTGTTGTCATACTTCTCATGG-3’.
- LOC100912399: upstream 5’-TAGACGTGCTCTCTCCCGCTTT-3’, downstream 5’-CCGGCTATGCAAAAGGAC-3’.
- p38MAPK: upstream 5’-ACATCGTGAAGTGCCAGAAT-3’, downstream 5’-GGCTTTAGGTCCCTGTGAA-3’.

### Lentiviral transfection

Lentiviral vectors were used for LOC100912399 overexpression, LOC100912399 knockdown, and p38MAPK overexpression. Preliminary experiments were conducted to determine the optimal transfection conditions. Third-passage BMSCs in good condition were seeded into 96-well culture plates at a density of 3 × 10³ cells per well and cultured for 8–12 h until cell confluence reached 20%–30%. The complete medium was replaced with fresh complete medium containing the appropriate amount of lentivirus, which was calculated based on the multiplicity of infection (MOI) and virus titer using the following formula (Eq. 1):

1 $${\mathrm{Virus}}\;{\text{volume }} = {\text{ }}{{\left( {{\mathrm{MOI}} \times {\mathrm{number}}\;{\mathrm{of}}\;{\mathrm{cells}}} \right)} \mathord{\left/ {\vphantom {{\left( {{\mathrm{MOI}} \times {\mathrm{number}}\;{\mathrm{of}}\;{\mathrm{cells}}} \right)} {{\mathrm{virus}}\;{\mathrm{titer}}}}} \right. \kern-\nulldelimiterspace} {{\mathrm{virus}}\;{\mathrm{titer}}}}{\text{ }}$$

After virus addition, cells were cultured for 8–12 h, and then the medium was replaced with complete medium containing 0.25% puromycin for selection and maintenance. Five days after infection, cells were passaged three times to obtain stable cell lines. The efficiency of virus infection was observed and verified using an inverted fluorescence microscope.

### Statistical analysis

All data were analyzed using SPSS 23.0 statistical software (Version IBM SPSS Statistics 23.0 www.ibm.com), and the results were expressed as the mean ± standard deviation (x̄ ± s). One-way ANOVA was used for comparisons between multiple groups, and the LSD-t test was used for pairwise comparisons between groups. A *P*-value < 0.05 was considered statistically significant.

## Results

### Extraction and culture of BMSCs

**Fig. 1:**
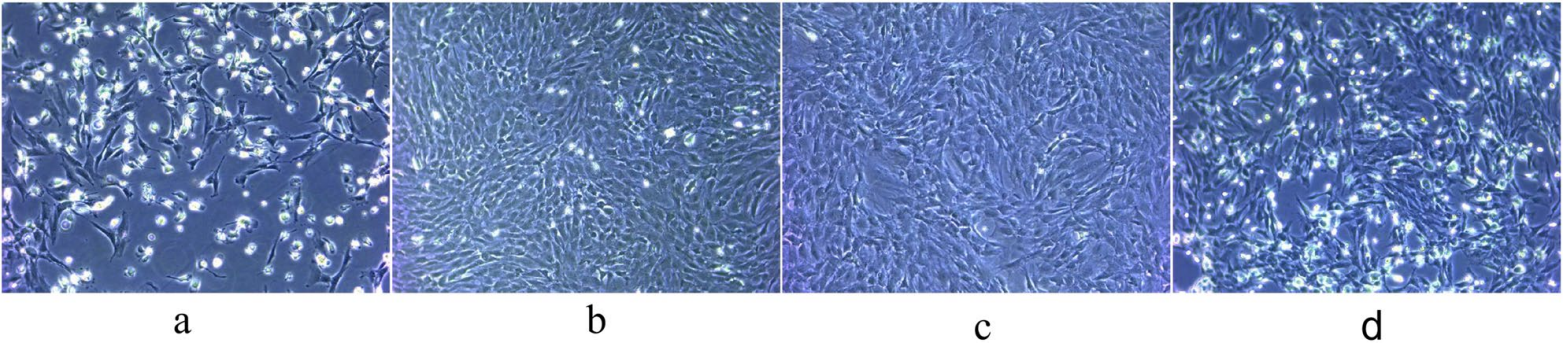
(**a**)–(**d**) Primary BMSCs cultured for 1 day (**a**) and 5 days (**b**); third-passage BMSCs (**c**) and resuscitated BMSCs (**d**) cultured for 3 days.

After 24 h of primary BMSC culture, the cells were basically adherent to the culture surface, with small and irregular morphologies (Fig. 1a). Approximately 5 days later, microscopic observation revealed that the cells grew adherently in a long spindle shape and gradually spread to cover the bottom of the culture flask (Fig. 1b). The digested and passaged cells were cultured to the third passage at a 1:3 split ratio; these cells were larger in size, predominantly spindle-shaped, and exhibited more active proliferation (Fig. 1c). Some apoptotic cells were observed after the resuscitation of third-passage cells, but the overall cell morphology remained unchanged (Fig. 1d).

### Flow cytometric detection of BMSC surface markers

**Fig. 2:**
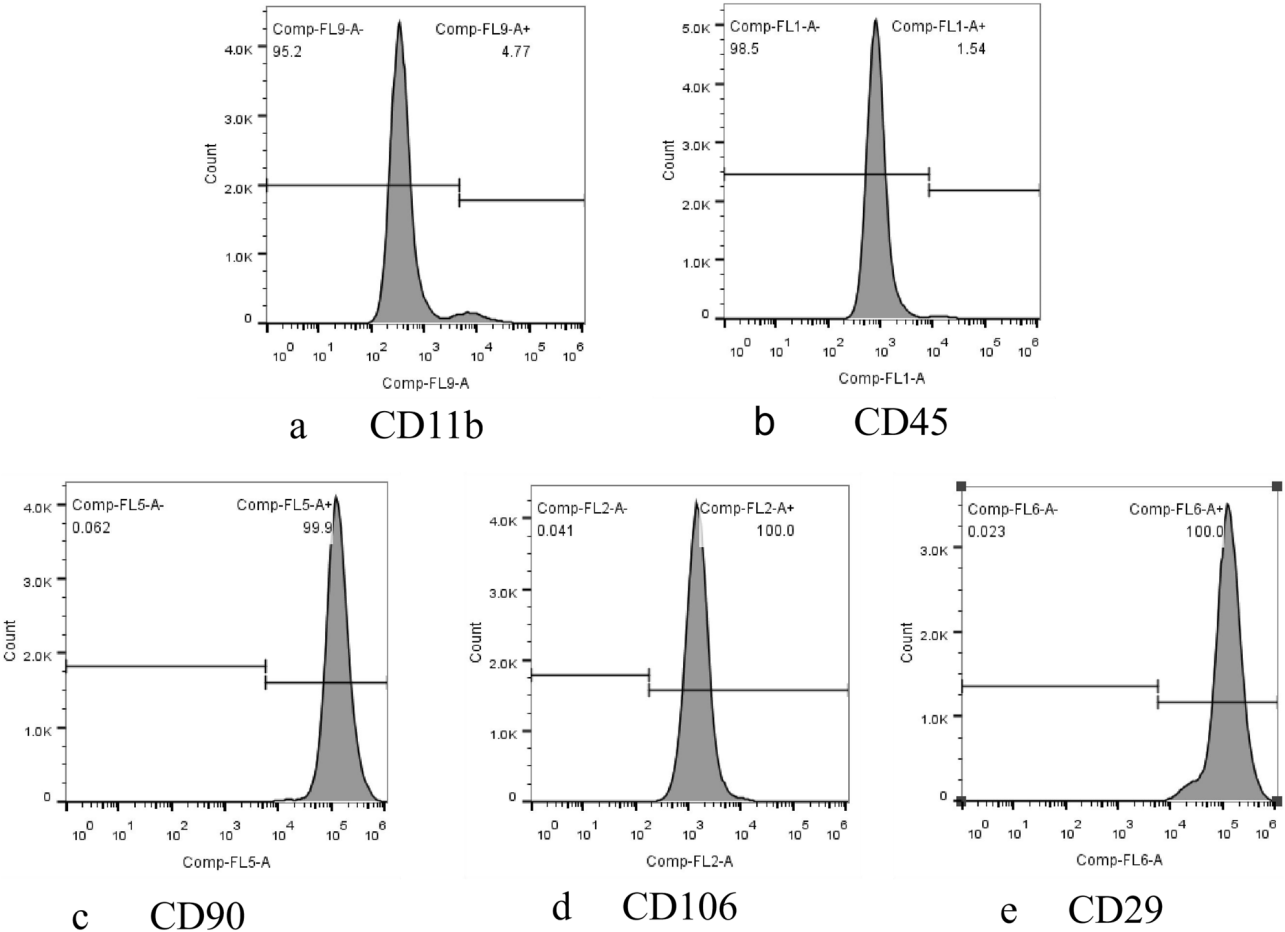
(**a**)–(**e**). Flow cytometric detection of surface antigen expression in third-passage BMSCs: CD11b (**a**), CD45 (**b**), CD90 (**c**), CD106 (**d**), and CD29 (**e**).

Flow cytometry was used to detect the expression of cell surface antigens (CD11b, CD45, CD90, CD106, CD29) in well-grown third-passage BMSCs.The analysis was performed using FlowJo software (Version FlowJo v10 www.flowjo.com). The results showed that the positive expression rates of these markers were all over 99% (Fig. 2a–e), confirming that the cultured BMSCs were of high purity and met the experimental requirements.

### Multilineage differentiation induction and identification of BMSCs

**Fig. 3:**
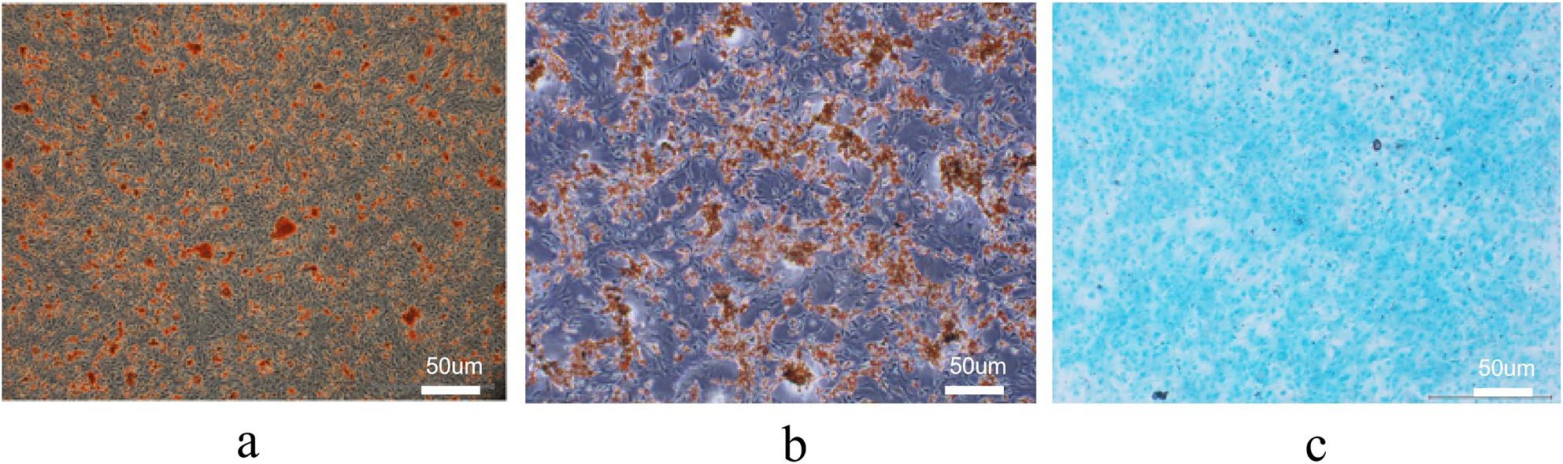
(**a**)–(**c**). Alizarin Red S staining (osteogenic differentiation,** a**), Oil Red O staining (adipogenic differentiation,** b**), and Alcian Blue staining (chondrogenic differentiation,** c**) under an inverted phase-contrast microscope (×100). Scale bars = 50 μm.

Third-passage BMSCs were digested, centrifuged, and resuspended at a density of 2 × 10⁵ cells/mL. The cell suspension was seeded into six-well plates, and when cell confluence reached 80%, the control group was maintained in complete medium, while the experimental groups were cultured in osteogenic, adipogenic, or chondrogenic differentiation media. The culture medium was changed every 2–3 days for 2–4 weeks. After induction, the cells were washed, and differentiation was identified by Alizarin Red S (osteogenic), Oil Red O (adipogenic), and Alcian Blue (chondrogenic) staining. Alizarin Red S staining revealed abundant red mineralized nodules in the cytoplasm; Oil Red O staining showed red lipid droplets; and Alcian Blue staining demonstrated blue proteoglycan deposition (Fig. 3a–c).

### Lentiviral transfection of LOC100912399 and P38MAPK

**Fig. 4:**
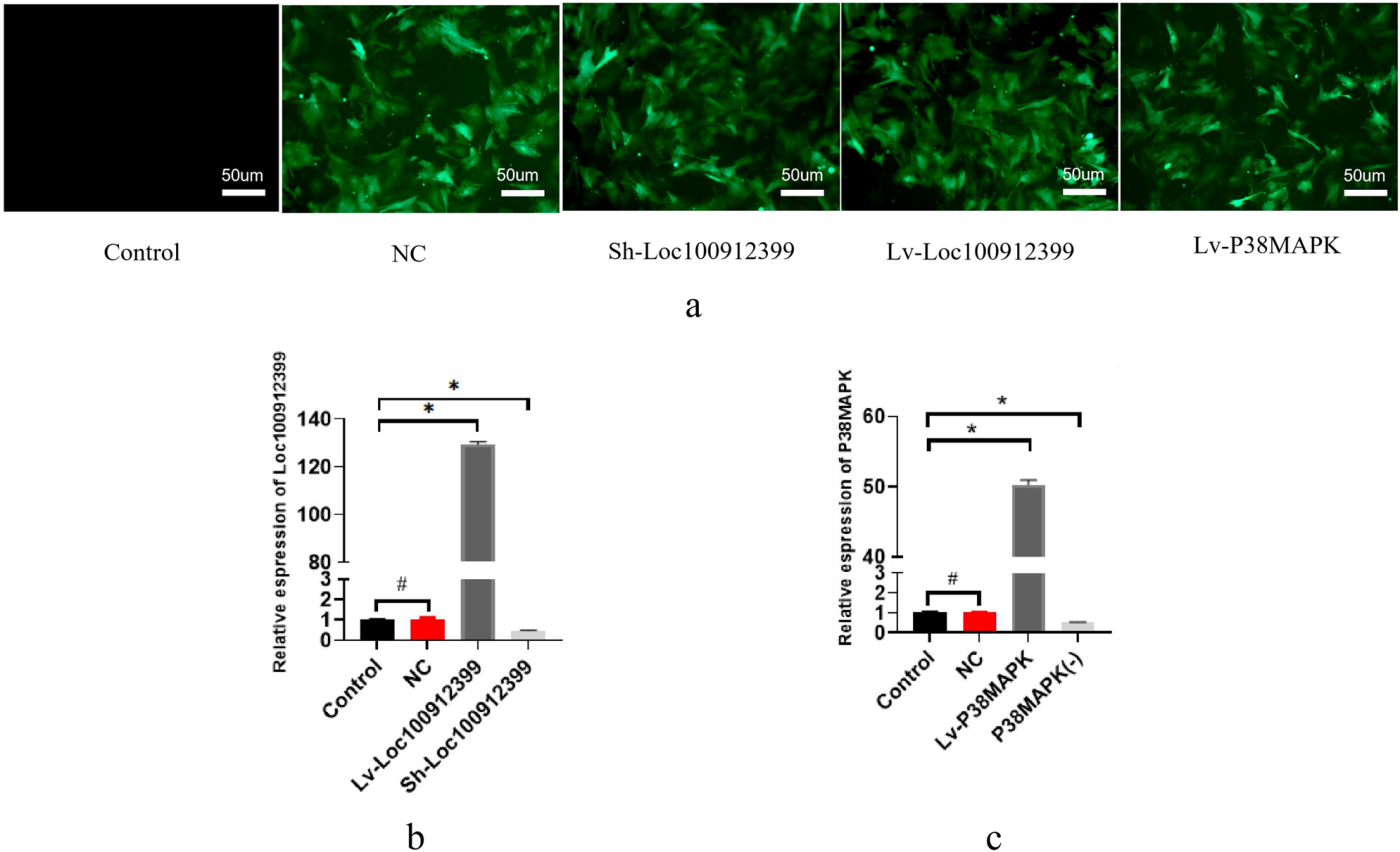
(**a**)–(**c**). Fluorescent observation of all lentiviral transfection groups (**a**); q-PCR detection of LOC100912399 expression (**b**) and P38MAPK expression (**c**). *P < 0.05, #*P* > 0.05.

Third-passage BMSCs were transfected with empty virus (NC), LOC100912399 knockdown lentivirus (Sh-LOC100912399), LOC100912399 overexpression lentivirus (Lv-LOC100912399), or P38MAPK overexpression lentivirus (Lv-P38MAPK), respectively. On day 5 post-transfection, fluorescent protein expression was observed in all lentiviral transfection groups under an inverted fluorescence microscope (Fig. 4a), indicating successful viral infection. q-PCR was performed to detect the expression levels of LOC100912399 and P38MAPK in each group(Data analysis: GraphPad Prism, Version 11.0.0, www.graphpad.com). There was no significant difference in LOC100912399 expression between the control group and the empty vector (NC) group (*P* > 0.05). Compared with the control group, LOC100912399 expression was significantly increased in the overexpression group and significantly decreased in the knockdown group (both *P* < 0.05) (Fig. 4b). Similarly, P38MAPK expression showed no significant difference between the control group and the NC group (*P* > 0.05), but was significantly increased in the P38MAPK overexpression group and significantly decreased in the P38MAPK inhibitor group compared with the control group (both *P* < 0.05) (Fig. 4c). These results confirm the success of LOC100912399 and P38MAPK lentiviral transfection.

### Establishment of an in vitro oxidative stress-induced osteoporosis model in BMSCs

**Fig. 5:**
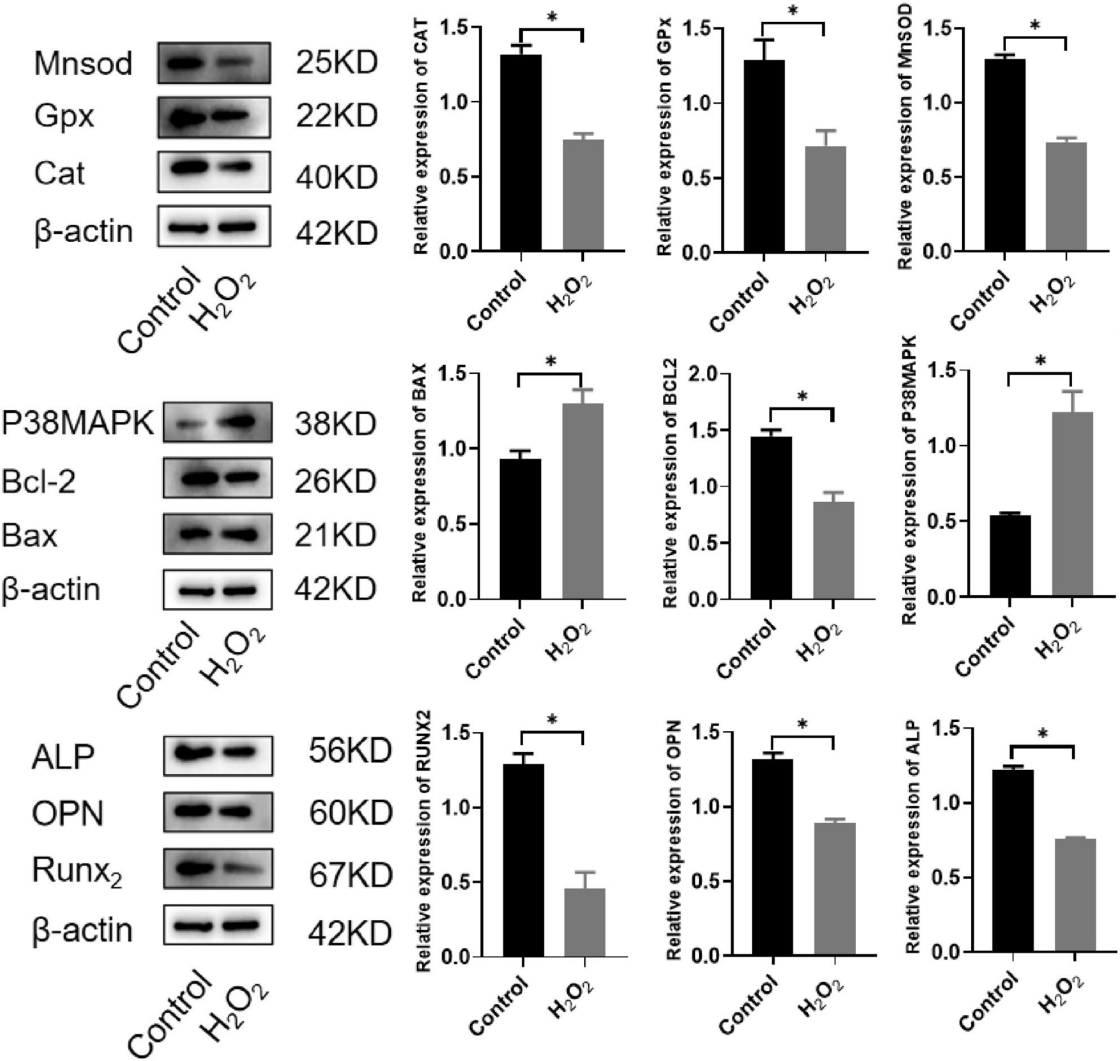
Establishment of an in vitro oxidative stress-induced osteoporosis model in BMSCs. Original blots/gels are presented in Supplementary Fig. 5.

Third-passage BMSCs were divided into control and oxidative stress (H_2_O_2_-treated) groups and cultured for 48 h. Western blot analysis was used to detect protein expression. Compared with the control group(ImageJ software, Version ImageJ2, imagej.net), the oxidative stress group showed decreased expression levels of antioxidant proteins (MnSOD, CAT, GPx) and the anti-apoptotic protein Bcl-2, while the expression of pro-apoptotic proteins (Bax, P38MAPK) was increased. Additionally, the expression of osteogenic markers (RUNX2, OPN, ALP) was reduced (all *P* < 0.05). These results indicate that oxidative stress promotes BMSC apoptosis and inhibits osteogenic differentiation, confirming the successful establishment of an in vitro oxidative stress-induced osteoporosis model in BMSCs (Fig. 5).

### Effects of the interaction between LOC100912399 and P38MAPK on BMSCs under oxidative stress conditions

On day 5 post-lentiviral infection, the expression levels of LOC100912399 and P38MAPK in BMSCs were confirmed to meet experimental requirements. The experiment was conducted in six groups: control (Control), oxidative stress (H_2_O_2_), LOC100912399 knockdown + oxidative stress (Sh-LOC100912399 + H_2_O_2_), LOC100912399 knockdown + P38MAPK overexpression + oxidative stress (Sh-LOC100912399 + P38 + H_2_O_2_), LOC100912399 overexpression + oxidative stress (Lv-LOC100912399 + H_2_O_2_), and LOC100912399 overexpression + P38MAPK inhibitor + oxidative stress (Lv-LOC100912399-P38 + H_2_O_2_). The aim was to investigate the effects of the interaction between LOC100912399 and P38MAPK on the oxidative stress response of BMSCs.

**Fig. 6:**
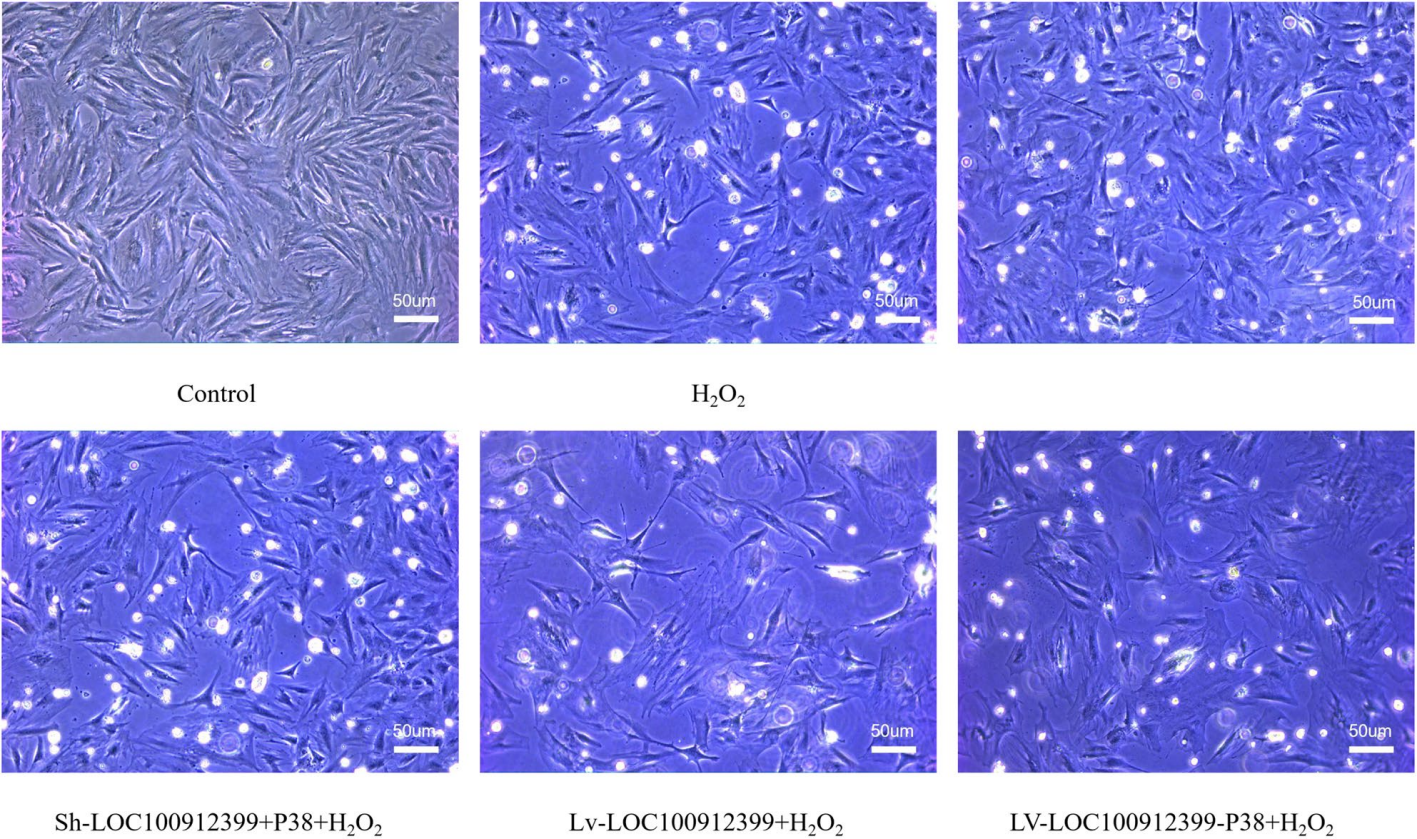
Morphology of BMSCs in each group observed under an inverted phase-contrast microscope (×100). Scale bars = 50 μm.

Cell morphology and quantity were observed under an inverted phase-contrast microscope. The control group showed good cell morphology, with spindle-shaped and flattened cells arranged regularly to cover the bottom of the flask. Compared with the control group, the oxidative stress group exhibited a reduced number of cells, irregular morphological changes, and shrunken, adherent, or suspended apoptotic/necrotic cells. The LOC100912399 knockdown + oxidative stress group had more regular cell morphology, with most cells being spindle-shaped and distributed regularly on the flask bottom; however, overexpression of P38MAPK on this basis increased the number of suspended cells (though morphology remained relatively regular). In the LOC100912399 overexpression + oxidative stress group, the number of adherent cells was significantly reduced compared with the oxidative stress group, and the cells were shrunken, shiny, or extremely irregular, with a large number of suspended apoptotic/necrotic cells. Reducing P38MAPK expression in this group decreased the number of suspended cells and improved cell morphology regularity (Fig. 6).

**Fig. 7:**
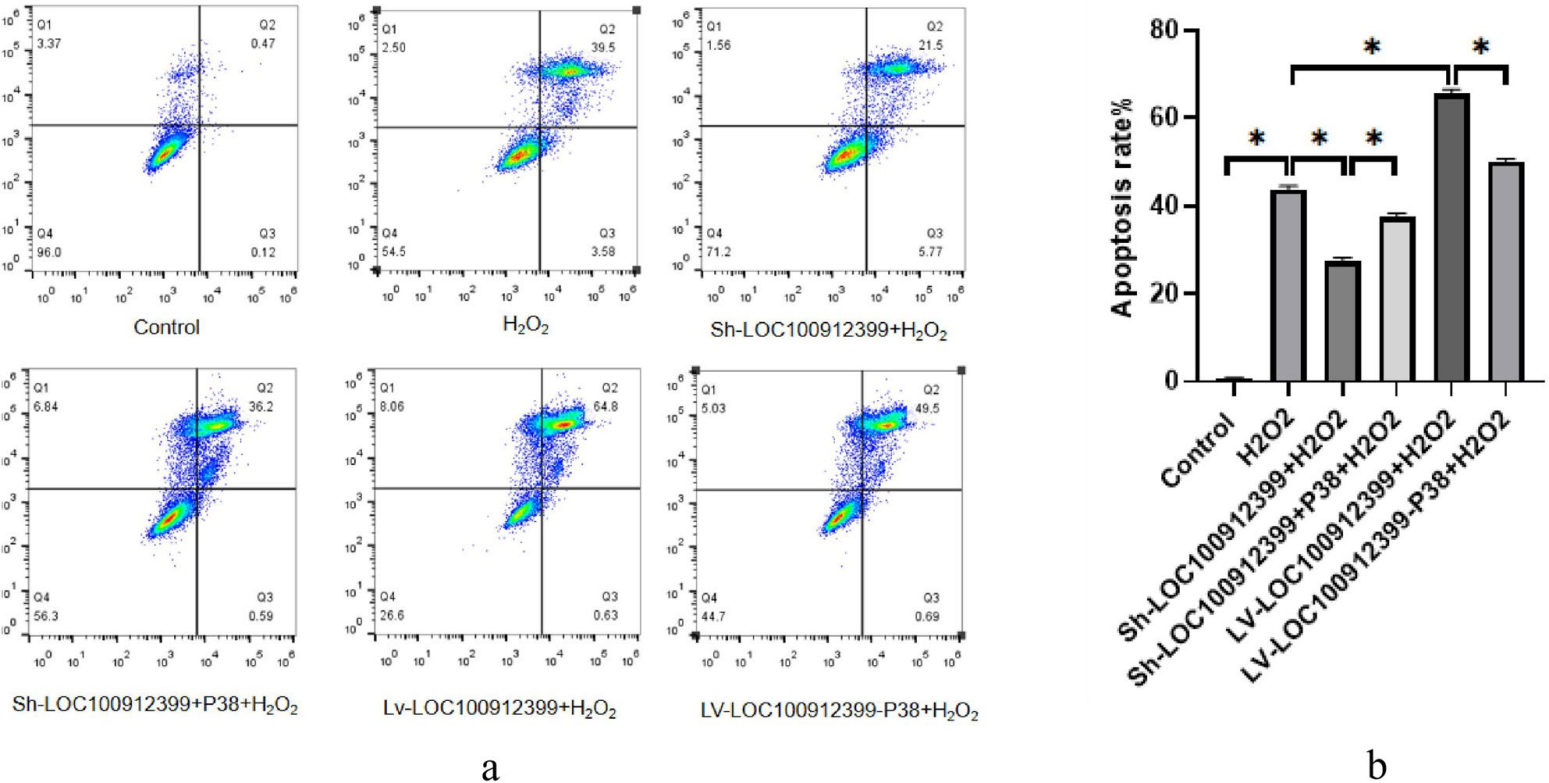
(**a**)–(**b**). Flow cytometric detection of BMSC apoptosis: representative flow cytometry plots (**a**) and apoptosis rate histogram (**b**). *P < 0.05.

Flow cytometric analysis showed that the apoptosis rate was significantly higher in the oxidative stress group than in the control group (*P* < 0.05). Compared with the oxidative stress group, the apoptosis rate was decreased in the LOC100912399 knockdown + oxidative stress group (*P* < 0.05), but increased when P38MAPK was overexpressed on this basis (*P* < 0.05). The apoptosis rate was significantly higher in the LOC100912399 overexpression + oxidative stress group than in the oxidative stress group (*P* < 0.05). These results suggest that overexpression of LOC100912399 promotes oxidative stress-induced apoptosis in BMSCs, while knockdown of LOC100912399 inhibits this process, and these effects may be mediated through the P38MAPK signaling pathway (Fig. 7a–b).

**Fig. 8:**
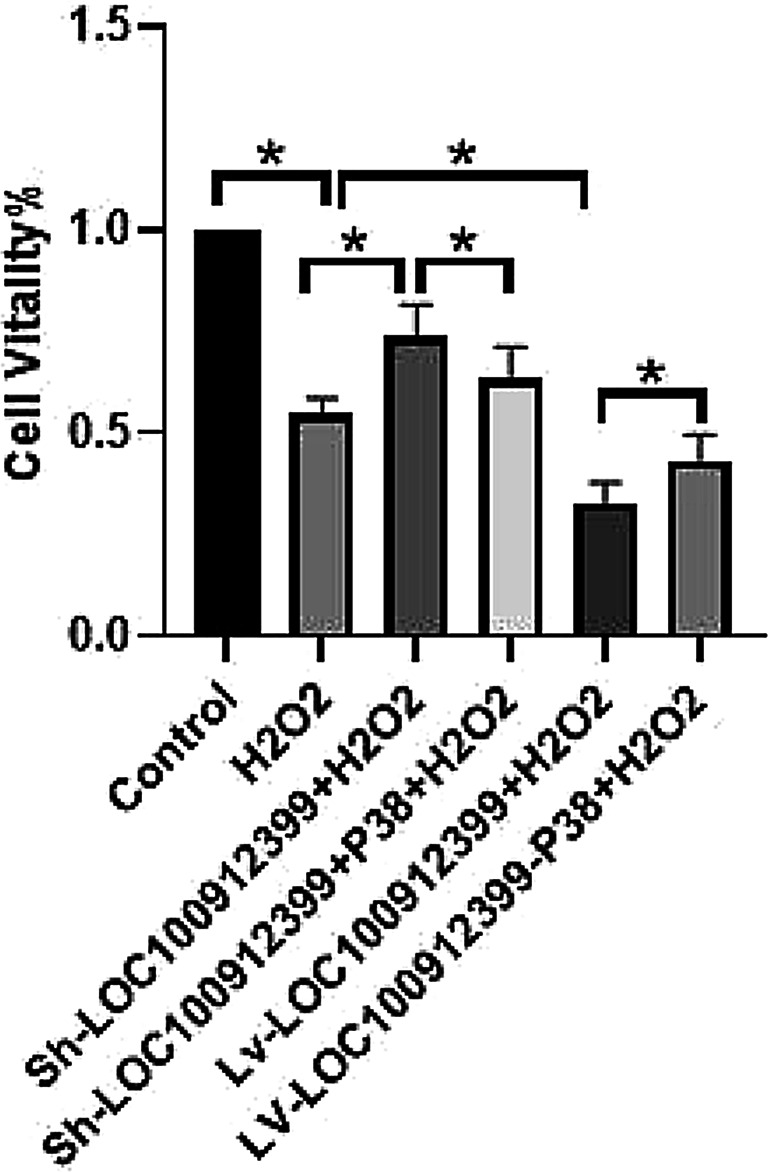
CCK-8 assay for cell viability in each group. *P < 0.05.

CCK-8 assay results showed that cell viability was significantly lower in the oxidative stress group than in the control group (*P* < 0.05). Compared with the oxidative stress group, cell viability was increased in the LOC100912399 knockdown + oxidative stress group (*P* < 0.05), but decreased when P38MAPK was overexpressed (*P* < 0.05). Cell viability was significantly reduced in the LOC100912399 overexpression + oxidative stress group compared with the oxidative stress group (*P* < 0.05), while downregulating P38MAPK expression increased cell viability (*P* < 0.05). These findings indicate that overexpression of LOC100912399 impairs BMSC viability, while knockdown of LOC100912399 enhances it, and the P38MAPK signaling pathway may be involved in mediating these effects (Fig. 8).

Western blot was used to detect the relative expression levels of oxidative stress-related proteins (MnSOD, CAT, GPx), apoptosis-related proteins (Bcl-2, Bax, P38MAPK), and osteogenic-related proteins (RUNX2, OPN, ALP) in each group. Compared with the oxidative stress group:

- The LOC100912399 knockdown + oxidative stress group showed significantly increased expression of antioxidant proteins (MnSOD, CAT, GPx), the anti-apoptotic protein Bcl-2, and osteogenic markers (RUNX2, OPN, ALP), along with decreased expression of pro-apoptotic proteins (Bax, P38MAPK) (all *P* < 0.05); these effects were reversed by P38MAPK overexpression (all *P* < 0.05).
- The LOC100912399 overexpression + oxidative stress group showed significantly decreased expression of antioxidant proteins (MnSOD, CAT, GPx), the anti-apoptotic protein Bcl-2, and osteogenic markers (RUNX2, OPN, ALP), along with increased expression of pro-apoptotic proteins (Bax, P38MAPK) (all *P* < 0.05); these effects were reversed by inhibiting P38MAPK expression (all *P* < 0.05).

**Fig. 9:**
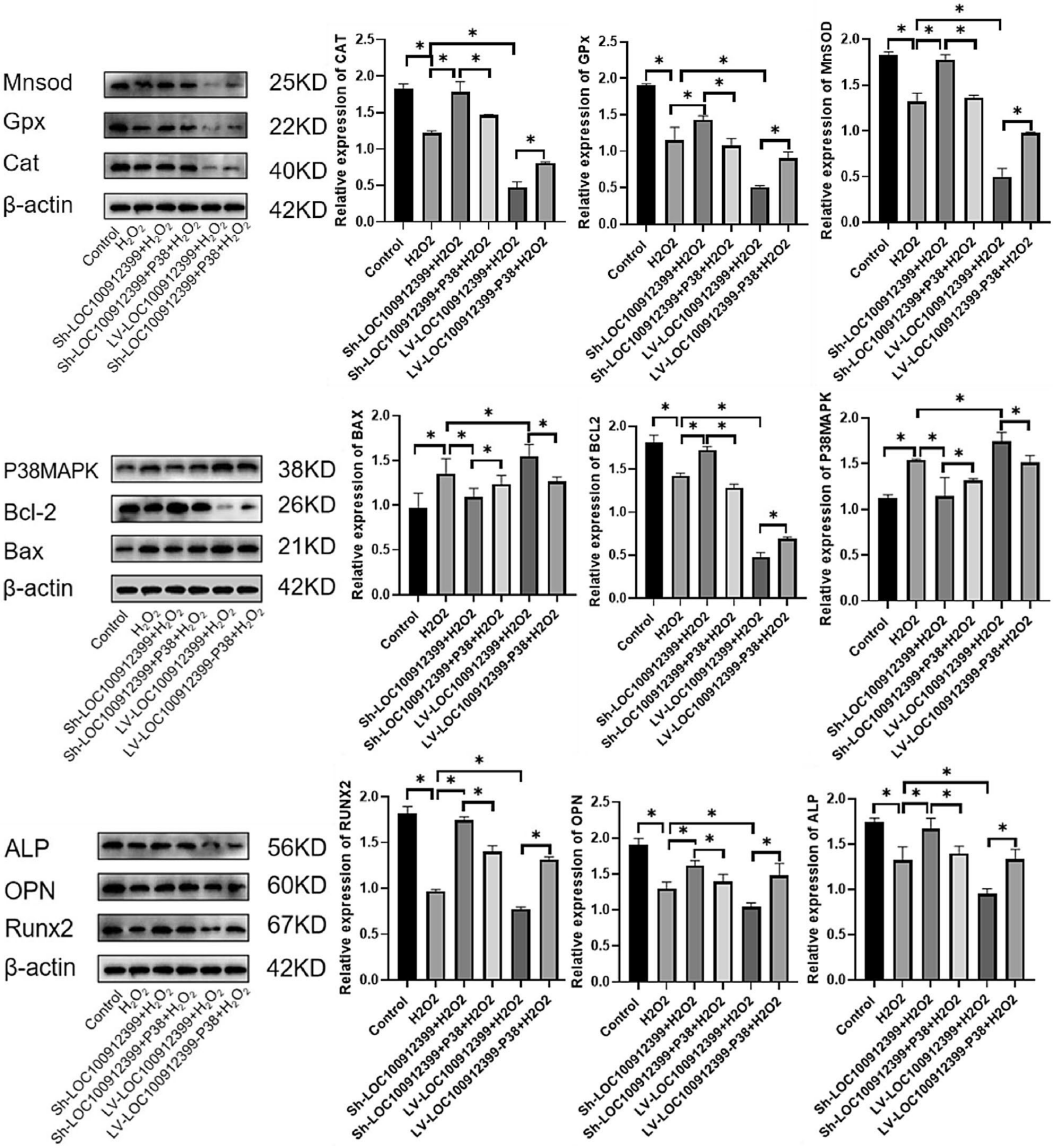
Relative expression levels of related proteins detected by Western blot. *P < 0.05. Original blots/gels are presented in Supplementary Fig. 9.

These results suggest that LOC100912399 may regulate H_2_O_2_-induced oxidative stress, apoptosis, and osteogenic differentiation in BMSCs through the P38MAPK signaling pathway (Fig. 9).

## Discussion

Oxidative stress (OS) arises when external stimuli disrupt intracellular redox homeostasis, leading to excessive accumulation of reactive oxygen species (ROS) and reactive nitrogen species (RNS) while impairing cellular scavenging capacity^1^. This imbalance causes damage to DNA, proteins, and lipids, thereby triggering the activation of apoptotic signaling pathways^1^. Osteoporosis (OP) is a systemic metabolic bone disorder characterized by an imbalance between osteoblast-mediated bone formation and osteoclast-mediated bone resorption^20^. Accumulating evidence identifies OS as an independent risk factor for OP, as it impairs the differentiation and survival of bone marrow mesenchymal stem cells (BMSCs), reduces osteoblast numbers, and hyperactivates osteoclasts—ultimately resulting in decreased bone mass and compromised bone strength^2^.

Manganese superoxide dismutase (MnSOD), catalase (CAT), and glutathione peroxidase (GPx) are key antioxidant enzymes that protect cell membranes, mitigate peroxide-induced damage, and counteract OS, serving as critical indicators of cellular senescence and apoptotic susceptibility^21^. Bcl-2-associated X protein (Bax), p38 mitogen-activated protein kinase (p38MAPK), and B-cell lymphoma 2 (Bcl-2) are core regulators of apoptosis, while runt-related transcription factor 2 (RUNX2), osteopontin (OPN), and alkaline phosphatase (ALP) are osteogenic-specific markers whose high expression promotes bone formation^22,23^. In the present study, hydrogen peroxide (H_2_O_2_)-induced OS was shown to trigger BMSC apoptosis, inhibit cell proliferation, and suppress osteogenic differentiation—validating the successful establishment of an in vitro OP model.

Long non-coding RNAs (lncRNAs) are stably expressed not only in tumor patients but also in the plasma, serum, and tissues of individuals with non-tumor diseases, underscoring their potential as diagnostic and therapeutic targets^24^. For instance, Xiao et al.^25^ demonstrated that lncRNA H19 is significantly downregulated in postmenopausal OP, and its reduced expression exacerbates the disease by upregulating miR-19b-3p to inhibit BMSC proliferation and osteogenic differentiation. Similarly, lncRNA WT1-AS is upregulated in OP and promotes osteoblast apoptosis through interaction with p53^26^. Consistent with these findings, our results reveal that LOC100912399 knockdown enhances BMSC antioxidant capacity, suppresses apoptosis, and promotes osteogenic differentiation. Specifically, LOC100912399 knockdown significantly increased the expression of antioxidant proteins (MnSOD, CAT, GPx), the anti-apoptotic protein Bcl-2, and osteogenic markers (RUNX2, OPN, ALP), while decreasing the levels of pro-apoptotic Bax and p38MAPK. Notably, overexpression of p38MAPK reversed these protective effects, indicating that LOC100912399 modulates OS-induced BMSC apoptosis and differentiation in a p38MAPK-dependent manner.

The p38MAPK pathway plays a pivotal role in regulating inflammation, cell differentiation, growth, and death^27^, and its aberrant activation is closely linked to OP pathogenesis. Pan et al.^28^ reported that downregulation of miR-182-5p in OP rats promotes osteoblast proliferation by activating the Rap1/MAPK pathway, while lncRNA AK125437 exacerbates OP by enhancing osteoclast activity through MAPK pathway activation^29^. The precise position of LOC100912399 within the p38MAPK signaling cascade—whether upstream or downstream—requires further validation. Future studies could employ more in-depth mechanistic approaches, such as detecting phosphorylated p38 (p-p38) protein levels or using specific p38 agonists/inhibitors. For example, if LOC100912399 overexpression significantly reduces p-p38 levels (with knockdown having the opposite effect), and modulating p38 activity does not alter LOC100912399 expression, this would support LOC100912399 functioning as an upstream regulator that promotes osteogenic differentiation by inhibiting p38MAPK phosphorylation.

This study has several limitations. First, the H_2_O_2_ concentration for OS induction was determined based on cytotoxicity assays, without direct quantitative measurement of intracellular ROS levels using fluorescent probes (e.g., DCFH-DA or MitoSOX). While the selected concentration effectively induced cellular stress responses and supported subsequent functional experiments, the lack of direct ROS detection limits precise calibration of OS intensity. Future research will integrate these technologies to comprehensively validate and quantify the OS state. Nevertheless, the coordinated changes in antioxidant proteins (MnSOD, GPx, CAT) and apoptosis markers (Bax) observed in this study functionally corroborate the effectiveness of the OS model and the protective role of LOC100912399, ensuring the robustness of our core conclusions.

Our experimental data confirm that H_2_O_2_ treatment increases p38MAPK expression in BMSCs, and LOC100912399 overexpression further elevates p38MAPK levels (with knockdown having the opposite effect). Moreover, p38MAPK overexpression enhances the inhibitory effects of LOC100912399 on BMSC proliferation and differentiation, while p38MAPK pathway blockade attenuates these effects. Collectively, these findings indicate that LOC100912399 regulates the p38MAPK pathway to modulate antioxidant enzyme expression, enhance resistance to OS, suppress apoptosis, and promote osteogenic differentiation—positioning it as a potential therapeutic target for OP.

## Conclusions

This study systematically elucidates the regulatory role and underlying mechanism of the long non-coding RNA LOC100912399 in bone marrow mesenchymal stem cell (BMSC) osteogenic differentiation under oxidative stress conditions. Our findings demonstrate that LOC100912399 exerts a critical modulatory effect on BMSC function through the p38 mitogen-activated protein kinase (p38MAPK) signaling pathway, with distinct outcomes based on its expression level. Specifically, LOC100912399 knockdown significantly upregulates the expression of key antioxidant enzymes (MnSOD, CAT, GPx), thereby enhancing BMSCs’ ability to scavenge reactive oxygen species and resist oxidative stress-induced damage. Concomitantly, this knockdown promotes the expression of the anti-apoptotic protein Bcl-2, reduces the pro-apoptotic protein Bax level, suppresses BMSC apoptosis, and elevates the expression of osteogenic marker genes (RUNX2, OPN, ALP)—ultimately fostering osteogenic differentiation. In contrast, LOC100912399 overexpression exacerbates oxidative stress imbalance, accelerates apoptotic processes, and inhibits osteogenic differentiation of BMSCs. Critically, these effects are tightly regulated by the p38MAPK signaling pathway: overexpression of p38MAPK reverses the protective and pro-osteogenic effects of LOC100912399 knockdown, while inhibition of p38MAPK mitigates the detrimental consequences of LOC100912399 overexpression. Collectively, these results confirm that LOC100912399 is a key mediator of oxidative stress-induced BMSC dysfunction, and its regulatory effects are dependent on the p38MAPK signaling pathway. Given the pivotal role of oxidative stress in osteoporosis pathogenesis, targeting LOC100912399—either by inhibiting its overexpression or enhancing its downregulation—offers a promising therapeutic strategy for restoring BMSC function, promoting bone formation, and alleviating osteoporosis. This work not only enriches our understanding of the molecular mechanisms linking lncRNAs, oxidative stress, and bone metabolism but also provides a novel, evidence-based target for the prevention and treatment of osteoporosis and other bone disorders associated with oxidative stress.

## Supplementary Information

Below is the link to the electronic supplementary material.

Supplementary Material 1

## Data Availability

The datasets used and/or analysed during the current study are available from the corresponding author on reasonable request.

## Abbreviations

lncRNA: Long non coding RNA
BMSCs: Bone marrow mesenchymal stem cells
p38MAPK: p38 mitogen activated protein kinase
LvLOC100912399: LOC100912399 overexpression
ShLOC100912399: LOC100912399 knockdown
Lvp38MAPK: p38MAPK overexpression
OS: Oxidative stress
OP: Osteoporosis
