# Supplementary material for: LOC100912399 regulates osteogenic differentiation of bone marrow mesenchymal stem cells through modulating p38MAPK signaling-mediated oxidative stress and apoptosis
Source: Sci Rep. 2026 Apr 1;16:15375. doi: 10.1038/s41598-026-45292-9 (PMC13184319; doi:10.1038/s41598-026-45292-9)
Supplement: Supplementary file 1 — Supplementary Material 1 [file 41598_2026_45292_MOESM1_ESM.pdf]

Figure 5

MnSOD

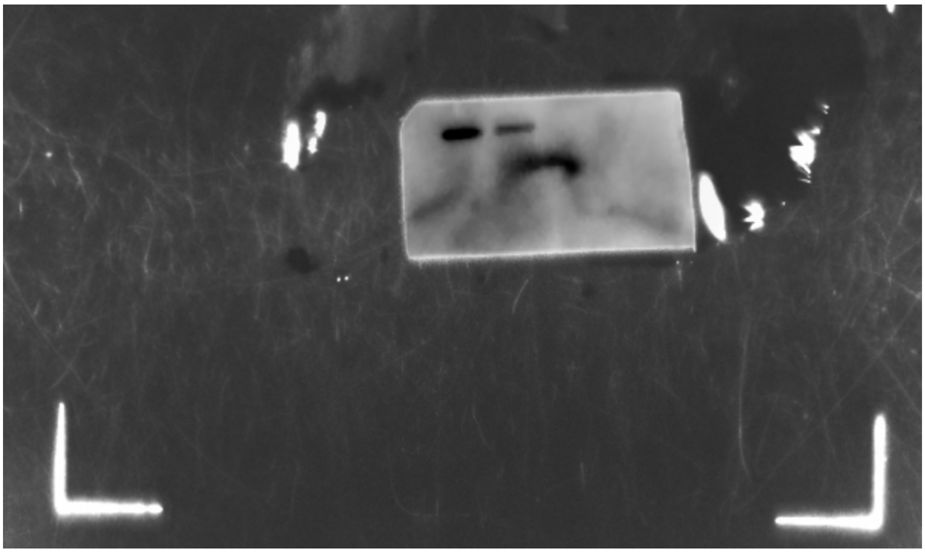

GPX

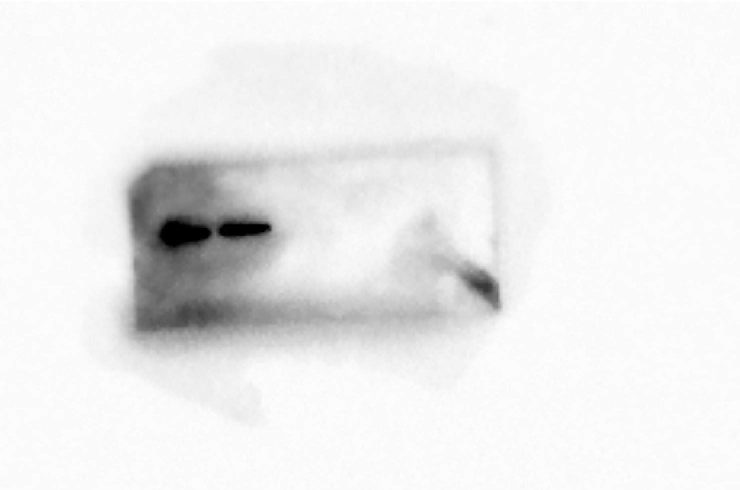

Cat

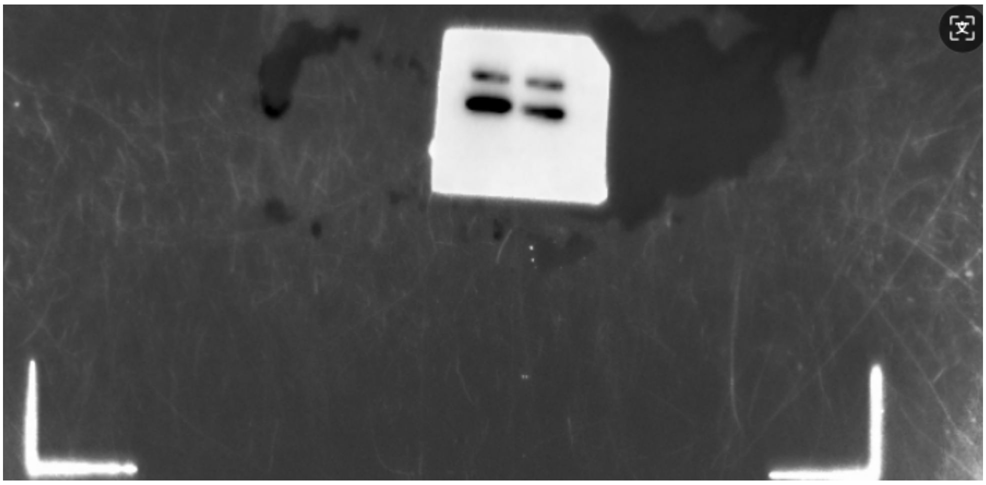

β -action

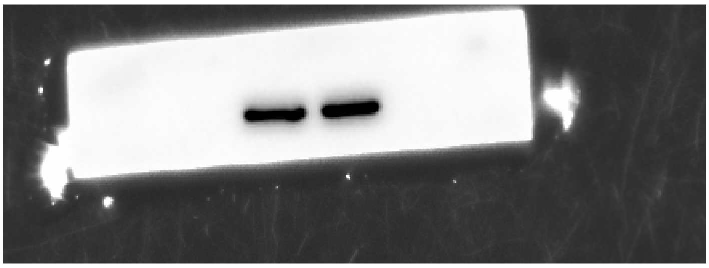

P38MAPK

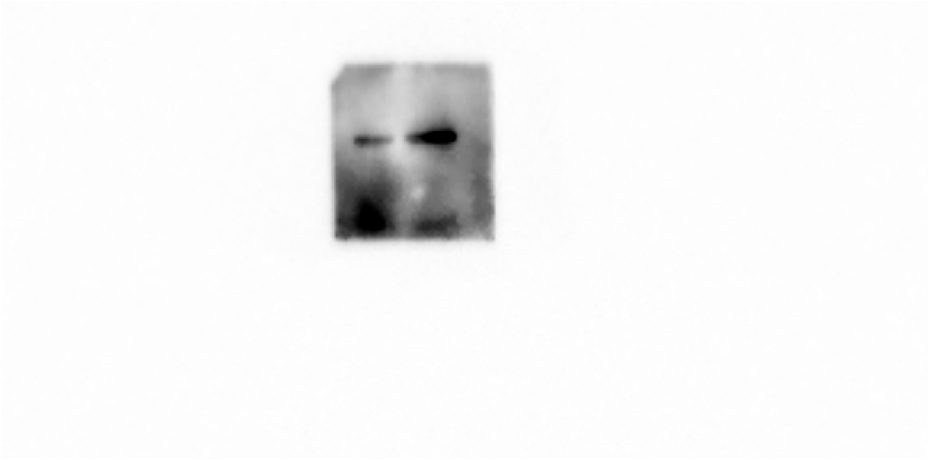

Bcl-2

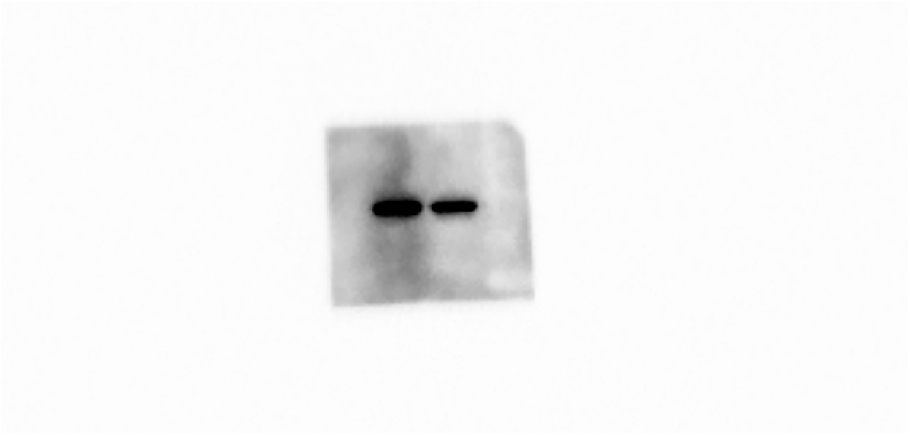

BAX

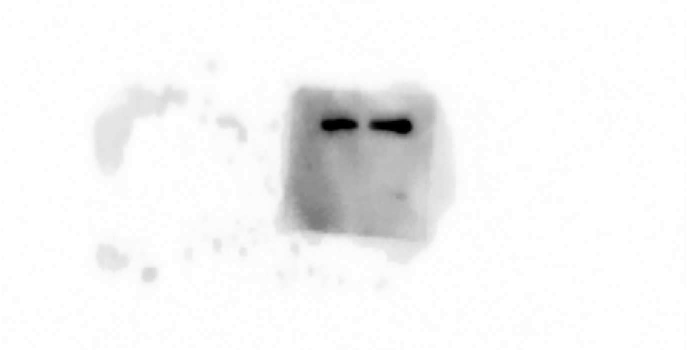

β -action

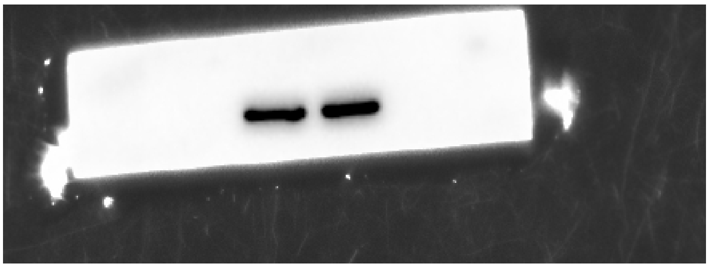

ALP

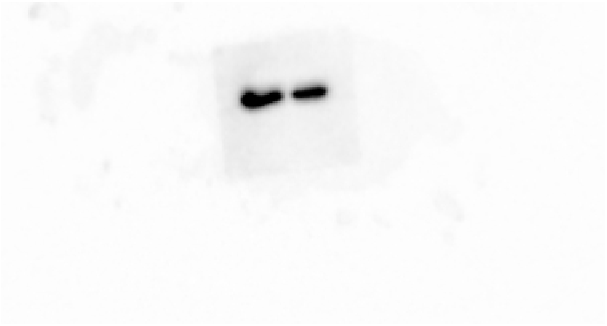

OPN

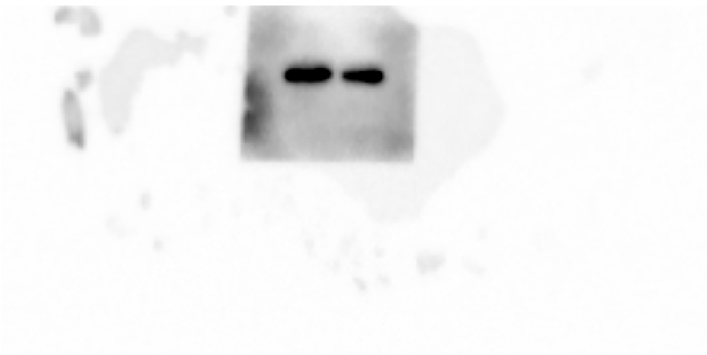

Runx2

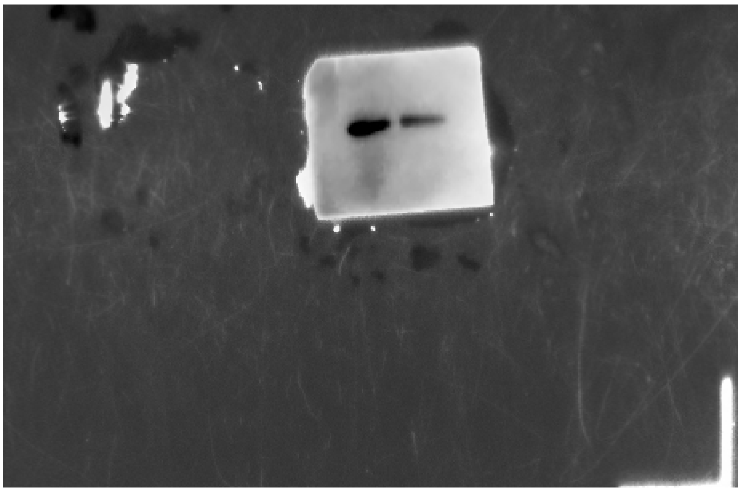

β -action

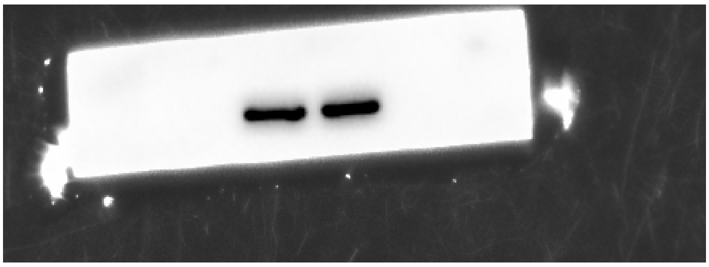

Figure 9

MnSOD

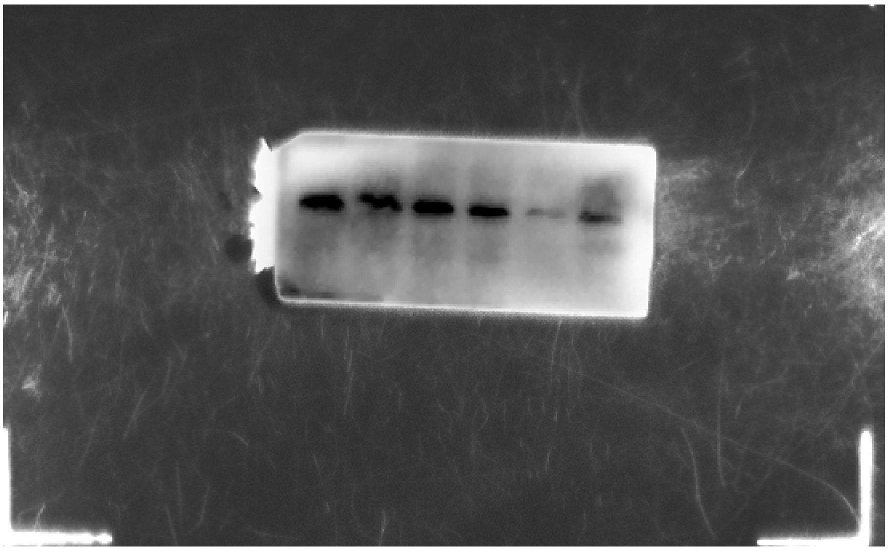

GPX

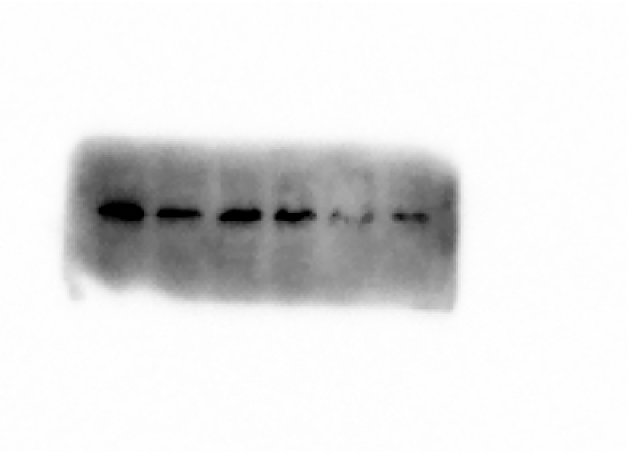

Cat

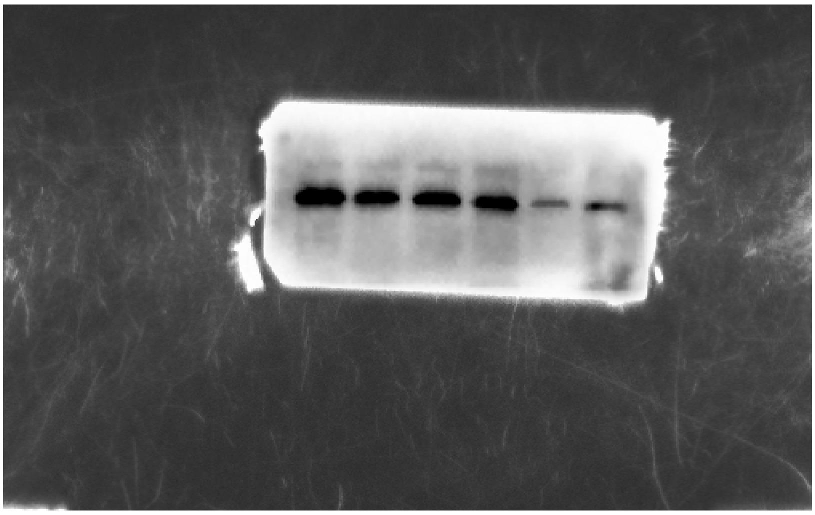

β -action

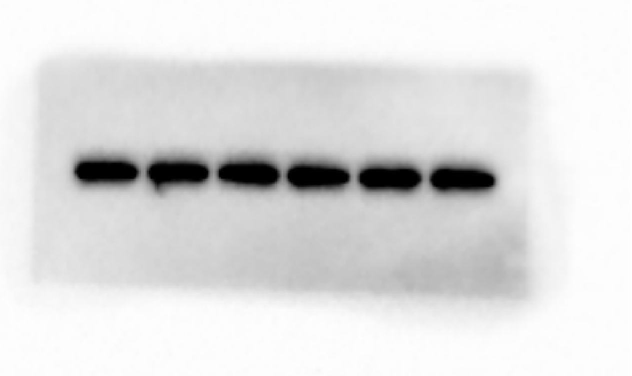

P38MAPK

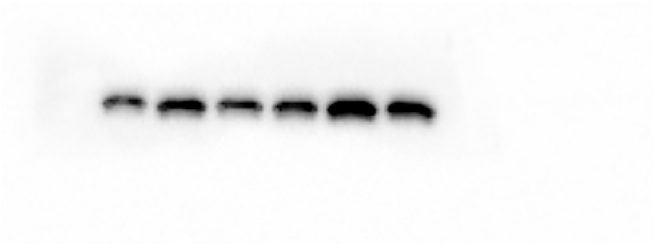

Bcl-2

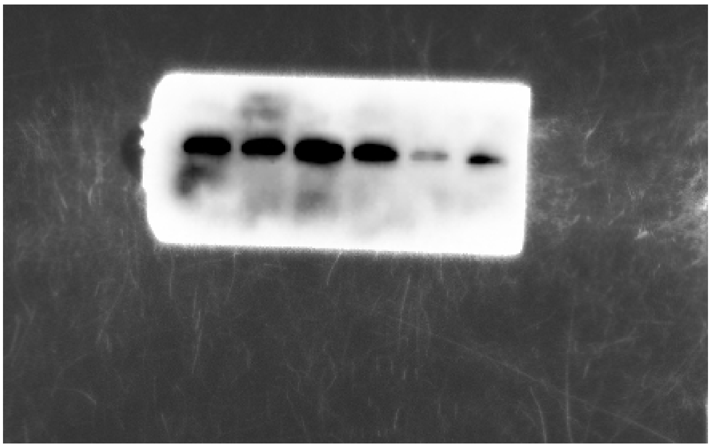

Bax

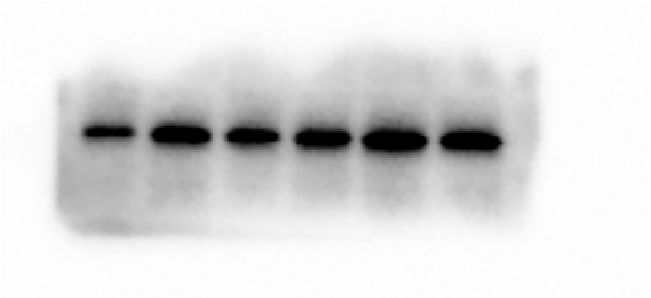

β -action

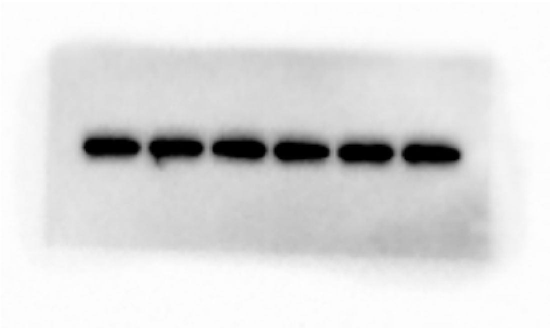

ALP

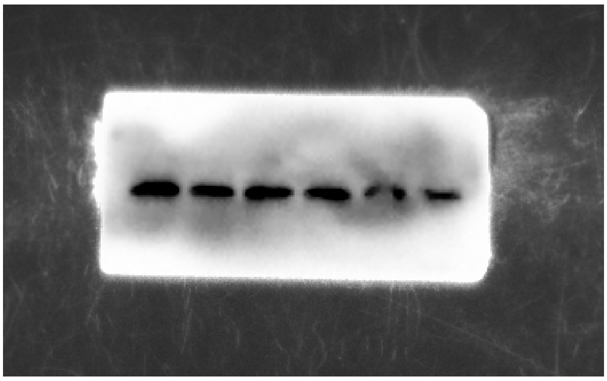

OPN

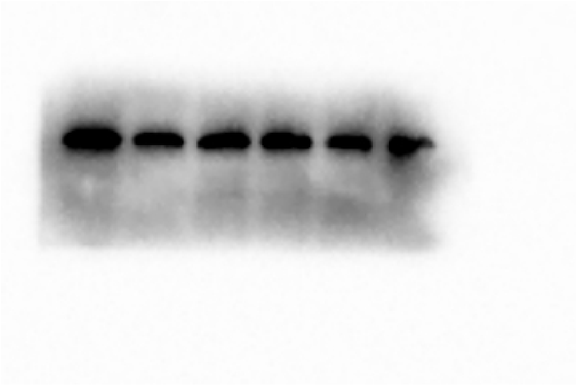

Runx2

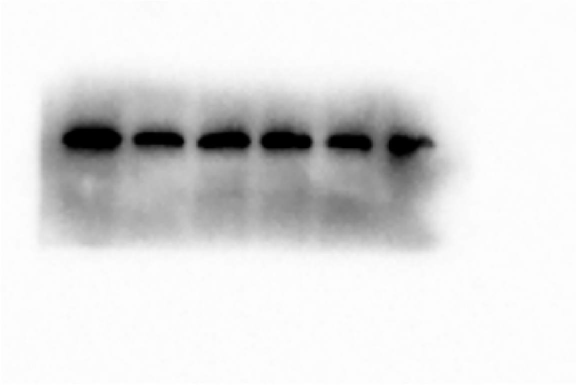

β -action

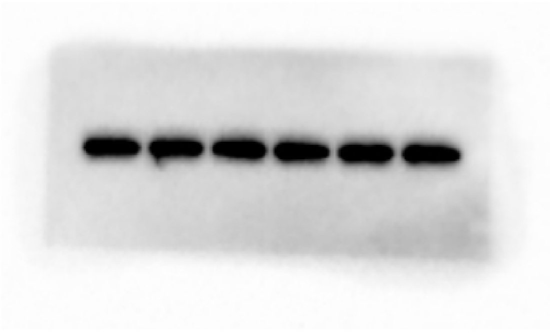

Hereby is declared that all samples were derived from the same experiment, and the gels/blots were processed in parallel.
